# *CYLD* as a key regulator of myocardial infarction-to-heart failure transition revealed by multi-omics integration

**DOI:** 10.3389/fgene.2025.1592985

**Published:** 2025-06-23

**Authors:** Jingya Xu, Zhonghua Dong, Zhaodong Li, Xuan Wang

**Affiliations:** ^1^ Department of Clinical Pharmacy, The First Affiliated Hospital of Shandong First Medical University and Shandong Provincial Qianfoshan Hospital, Shandong Engineering and Technology Research Center for Pediatric Drug Development, Shandong Medicine and Health Key Laboratory of Clinical Pharmacy, Jinan, China; ^2^ College of Pharmacy, Shandong University of Traditional Chinese Medicine, Jinan, China; ^3^ Guangdong Key Laboratory of Regional Immunity and Diseases, Department of Pathogen Biology, Shenzhen University School of Medicine, Shenzhen, China

**Keywords:** heart failure, myocardial infarction, WGCNA, machine learning, single-cell sequencing analysis, hub genes

## Abstract

**Introduction:**

Heart failure (HF) is the most common complication following myocardial infarction (MI) and frequently occurs during the postinfarction recovery phase. Despite the well-established association between HF and MI, the underlying molecular mechanisms driving their transition remain poorly understood.

**Methods:**

The aim of this study was to identify key regulatory genes involved in this transition via advanced computational tools. We conducted a comprehensive analysis of differentially expressed genes (DEGs) via Limma software, leveraging five independent datasets retrieved from the Gene Expression Omnibus (GEO) database: GSE59867, GSE62646, GSE168281, GSE267644, and GSE269269. Our multistep analytical pipeline included weighted gene coexpression network analysis (WGCNA) to map interacting genes, machine learning algorithms for robust classification, functional annotation via Kyoto Encyclopedia of Genes and Genomes (KEGG) to explore biological pathways, CIBERSORT correlation analysis linking hub genes with immune cell states, transcriptional regulation profiling of key hubs, and single-cell sequencing to assess the functional relevance of these hubs.

**Results:**

Our findings revealed that 413 DEGs were significantly different between MI and HF. WGCNA identified 98 genes associated with both conditions. Machine learning filtering further prioritized 10 hub genes: *GPER1*, *E2F5*, *DZIP3, CYLD*, *ADAMTS2*, *ZNF366*, *ST14, SNORD28*, *LHFPL2*, and *HIVEP2*. These hubs were significantly associated with immune-related processes, suggesting their potential role in the pathogenesis of HF after MI. Single-cell transcriptomic analysis demonstrated that *CYLD* exhibited the strongest correlation with the transition from MI to HF; using random forest modelling, we validated its predictive value in this context.

**Discussion:**

In conclusion, our study identified *CYLD* as a critical regulator of the transition from MI to HF. Our findings underscore the potential of hub genes as targets for novel therapeutic interventions aimed at mitigating MI-to-HF progression and improving patient outcomes.

## 1 Introduction

Myocardial infarction (MI) is a leading cause of morbidity and mortality worldwide, with heart failure (HF) being a common yet devastating complication (Bahit et al., 2018; Li et al., 2024). Despite advancements in treatment strategies, patients who experience MI remain at high risk for the development of HF, which significantly impacts their long-term prognosis (Kaul et al., 2013). HF frequently follows MI, and its prevalence among hospitalized patients with acute MI varies widely across studies, ranging from 14% to 36% (Bahit et al., 2018). HF significantly elevates the risk of mortality following MI. Over time, mortality rates after MI have decreased, primarily due to advancements in HF survival rates. This difference may be attributed to varying risk factors and underlying mechanisms that contribute to the development of HF at different time points following MI(Gerber et al., 2016; Jenca et al., 2021). Current guidelines recommend that patients undergo early risk assessment after acute MI to ensure the provision of appropriate therapy. Early and accurate risk stratification is crucial for identifying patients at risk of developing HF post-MI, as it guides treatment and improves prognosis (Ibanez et al., 2018; Collet et al., 2021; Jenca et al., 2021; Akhtar et al., 2024).

The American Heart Association and the European Society of Cardiology have long acknowledged the existence of an urgent public health need for the prevention of HF (Schocken et al., 2008; Ponikowski et al., 2014). The cohort of patients who have experienced an MI constitutes a high-risk demographic for the development of HF, rendering HF screening and prevention within this group particularly crucial. Failure to diagnose HF promptly or accurately can jeopardize patient outcomes and increase treatment expenses (Faridi et al., 2016; Jenca et al., 2021). If a biomarker is causally associated with HF, it may play a pivotal role in predicting the onset and progression of HF post-MI. This predictive capability not only aids in early diagnosis but also facilitates personalized treatment strategies; thus, these biomarkers may ultimately serve as a important targets for intervention. By identifying and targeting such biomarkers, healthcare providers can implement more effective preventive measures and therapeutic interventions, thereby improving patient outcomes and reducing the overall burden of HF. In pursuit of this goal, the identification of potential target genes involved in the development of HF post-MI is of paramount importance.

To further elucidate the molecular mechanisms driving HF after MI, our study employed a comprehensive bioinformatics approach. Given the swift advancements in microarray and high-throughput sequencing technologies, bioinformatics approaches are frequently employed in disease research. Using weighted gene coexpression network analysis (WGCNA), we identified several modules enriched for immune-related processes that were significantly associated with the transition from MI to HF. These findings were validated via the CIBERSORT algorithm, which revealed specific signatures linked to unfavourable outcomes in high-risk patients. Single-cell transcriptomic data provided additional insights into the heterogeneity of HF progression.

Our findings underscore the importance of early and accurate risk stratification for HF development in MI survivors. By identifying biomarkers that are causally associated with HF, we can develop targeted therapeutic strategies aimed at reversing or preventing disease progression.

## 2 Materials and methods

### 2.1 Data collection

In this study, we accessed gene expression microarray data from the Gene Expression Omnibus (GEO) database (http://www.ncbi.nlm.nih.gov/geo). The microarray data were obtained via multiple platforms: GPL6244 with accession numbers GSE59867 (Maciejak et al., 2015) and GSE62646 (Kiliszek et al., 2012), an additional external validation set GPL11154 with accession number GSE168281, and single-cell gene expression datasets GSE267644 (Kneuer et al., 2024) and GSE269269 (Qian et al., 2024).

### 2.2 Identification of differentially expressed genes (DEGs)

The initial expression matrix was normalized and processed via R software (version 4.4.1). To identify DEGs between the datasets GSE59867 and GSE62646, we utilized the “limma” package within the R environment. DEGs were selected based on an adjusted *P* value <0.05 and a fold-change threshold of |log FC| ≥ 0.5.

### 2.3 Construction of the WGCNA network

WGCNA is a widely used bioinformatics tool for revealing gene coexpression patterns across multiple samples by clustering genes with similar expression profiles into distinct modules. These gene modules can be further correlated with external traits or phenotypes to explore potential functional associations (Langfelder and Horvath, 2008). In this study, WGCNA was performed via the R WGCNA package. To construct a reliable coexpression network, we selected a soft-thresholding power of 11 based on the criterion for achieving a scale-free topology. Specifically, we evaluated a range of power values and identified the lowest power for which the scale-free topology fit index (*R* ^2^) exceeded 0.80 while ensuring relatively high mean connectivity. This approach aligns with the principle that biological networks tend to exhibit scale-free properties.

### 2.4 Identification of shared genes

To identify the core common genes, we performed a comprehensive analysis by first constructing Venn diagrams of genes identified through WGCNA and DEG analyses to identify overlapping genes. These shared genes were then selected for subsequent analysis.

### 2.5 Screening hub genes via the machine learning algorithm

Following the identification of shared genes, we applied two complementary machine learning algorithms to identify potential hub genes with the highest discriminative power. First, we used support vector machine recursive feature elimination (SVM-RFE), which employs a linear kernel function to ensure model interpretability and computational efficiency (Huang et al., 2014). Feature ranking was performed iteratively, and the optimal subset was selected based on the minimal cross-validation error. Next, the least absolute shrinkage and selection operator (LASSO) algorithm was implemented via the glmnet package in R (Li and Sillanpaa, 2012). We applied 10-fold cross-validation to identify the optimal value of the regularization parameter lambda (λ), defined as the value minimizing the mean cross-validated error (lambda.min). To validate the classification performance of the selected features, we constructed multiple supervised learning models—logistic regression (LR), linear discriminant analysis (LDA), k-nearest neighbour (KNN), support vector machine (SVM), random forest (RF), ridge regression, elastic net, naïve Bayes and XGBoost. Their predictive performance was evaluated via the area under the curve (AUC), sensitivity, specificity, positive predictive value (PPV) and negative predictive value (NPV). The model yielding the best overall metrics was adopted for hub gene selection.

### 2.6 Correlation analysis between infiltrating immune cells and hub genes

The CIBERSORT algorithm was employed for immune cell infiltration analysis. Spearman correlation analysis was performed to examine the relationships between infiltrating immune cells and hub genes, and the corrplot tool in R was used for visualization.

### 2.7 Candidate biomarker expression levels and diagnostic value

The ggplot2 package in R was used to analyse the expression levels of candidate biomarkers. To assess their potential diagnostic performance, receiver operating characteristic (ROC) analysis was conducted, with the AUC serving as a key metric for evaluating the efficacy of these markers.

### 2.8 Construction of the nomogram model

Based on the selected explanatory variables, we constructed a nomogram model employing the “rms” package. We calculated every item’s score by projecting upwards on a small scale (points) based on the characteristics of each variable of the patient. The total value was calculated by adding the scores of each item. A higher total value indicates a higher probability of HF. To assess the model’s accuracy, we conducted an analysis of the calibration curve, decision curve analysis (DCA) curve and clinical impact curve (Iasonos et al., 2008).

### 2.9 Single-cell sequencing analysis

The data were first subjected to standardized preprocessing via the Seurat software package (Stuart et al., 2019). The t-SNE method was subsequently applied to model spatial relationships between cellular clusters. Cluster annotation was then performed via the celldex tool, with particular emphasis on key functional cell types. By implementing the FindAllMarkers function combined with a log2(FC). In the threshold thresholding approach, significant marker genes for individual cell subsets were identified from the single-cell expression data.

## 3 Results

### 3.1 Elucidating key regulatory genes in the MI-to-HF pathway via DEGs and WGCNA

To identify the key regulatory genes associated with progression from MI-to-HF, we performed a genome-wide gene expression analysis using publicly available datasets from the GEO database (GSE59867 and GSE62646). These datasets compare gene expression profiles between HF patients and those who remained stable post-MI.

**FIGURE 1:**
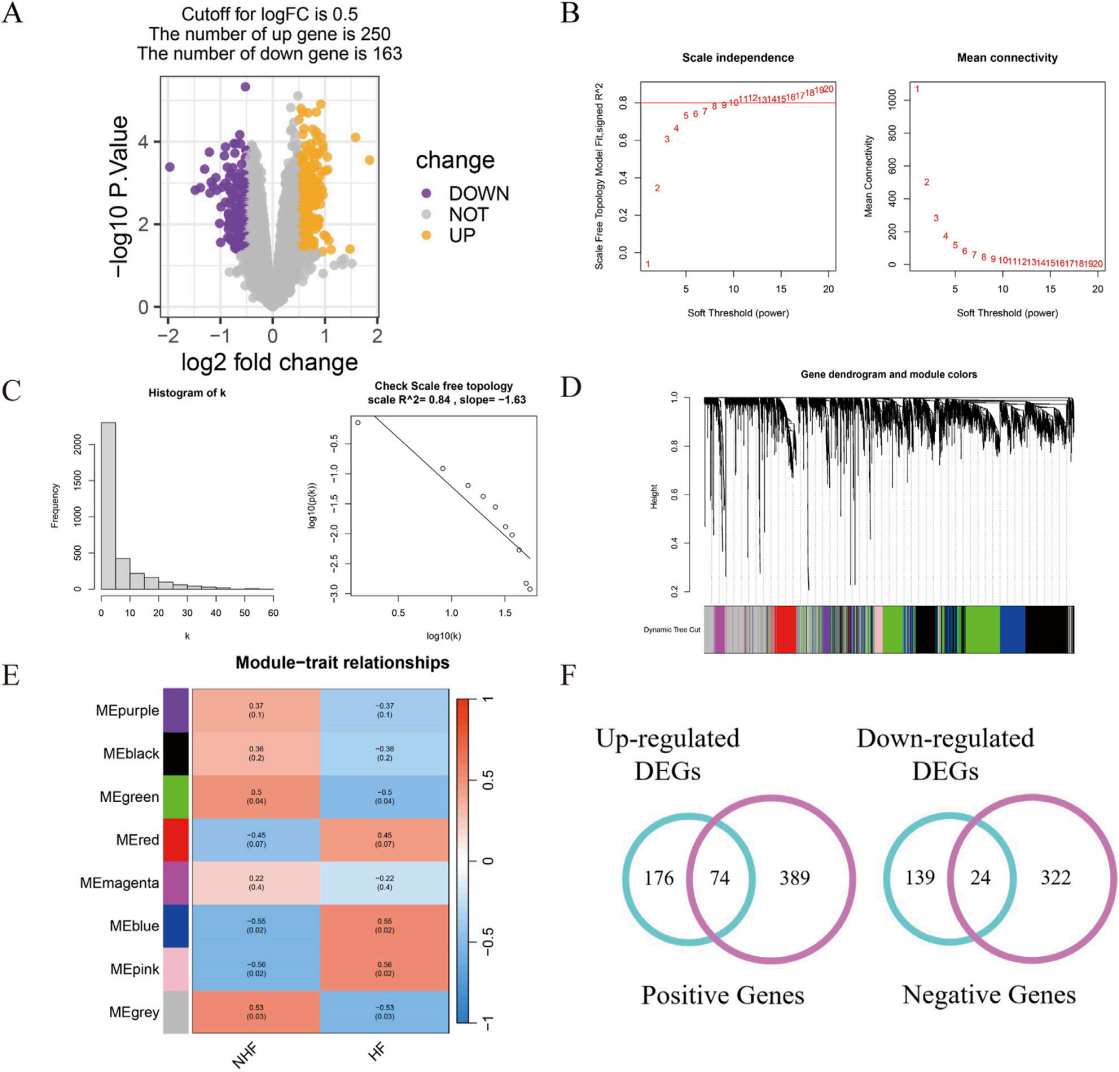
Identification of differentially expressed genes. **(A)** Differential expression analysis between MI patients with HF and non-HF; **(B)** A scale-free network model was utilized to identify the optimal β value; **(C)** Network connectivity distribution analysis revealed a right-skewed histogram pattern; **(D)** WGCNA was employed to construct modules via hierarchical clustering; **(E)** A heat map was generated to visualize the association between WGCNA modules and enrollment characteristics; **(F)** Venn diagrams were used to illustrate the overlap of DEGs associated withincident HF risk across patient cohorts.

The raw data were normalized via the “limma” package (Supplementary Figure S1), and DEGs were identified under stringent criteria (*P* value <0.05 and log-fold change |logFC| ≥ 0.5). This analysis revealed 413 DEGs, including 250 upregulated and 163 downregulated genes (Figure 1A).

For network construction, we employed the WGCNA approach. A soft threshold of 11 was selected to ensure network scalability (Figures 1B,C), resulting in the identification of eight distinct modules, each represented by a unique colour (Figure 1D; Supplementary Figure S2A). Among the modules, the blue, pink, and green modules were significantly correlated with MI patients at high risk of HF progression (Supplementary Figure S2B–D). Specifically, the blue module exhibited a positive correlation (r = 0.55, *P* = 0.02), as did the pink module (r = 0.56, *P* = 0.02), whereas the green module displayed a negative correlation (r = −0.5, *P* = 0.04), suggesting a potential protective role against HF progression (Figure 1E).

To identify candidate genes, we performed a Venn diagram analysis of the DEGs and module genes, which yielded 98 overlapping genes (Figure 1F). These genes were identified as potential regulatory factors driving the MI-to-HF transition.

### 3.2 Hub gene identification and validation in MI-to-HF progression

**FIGURE 2:**
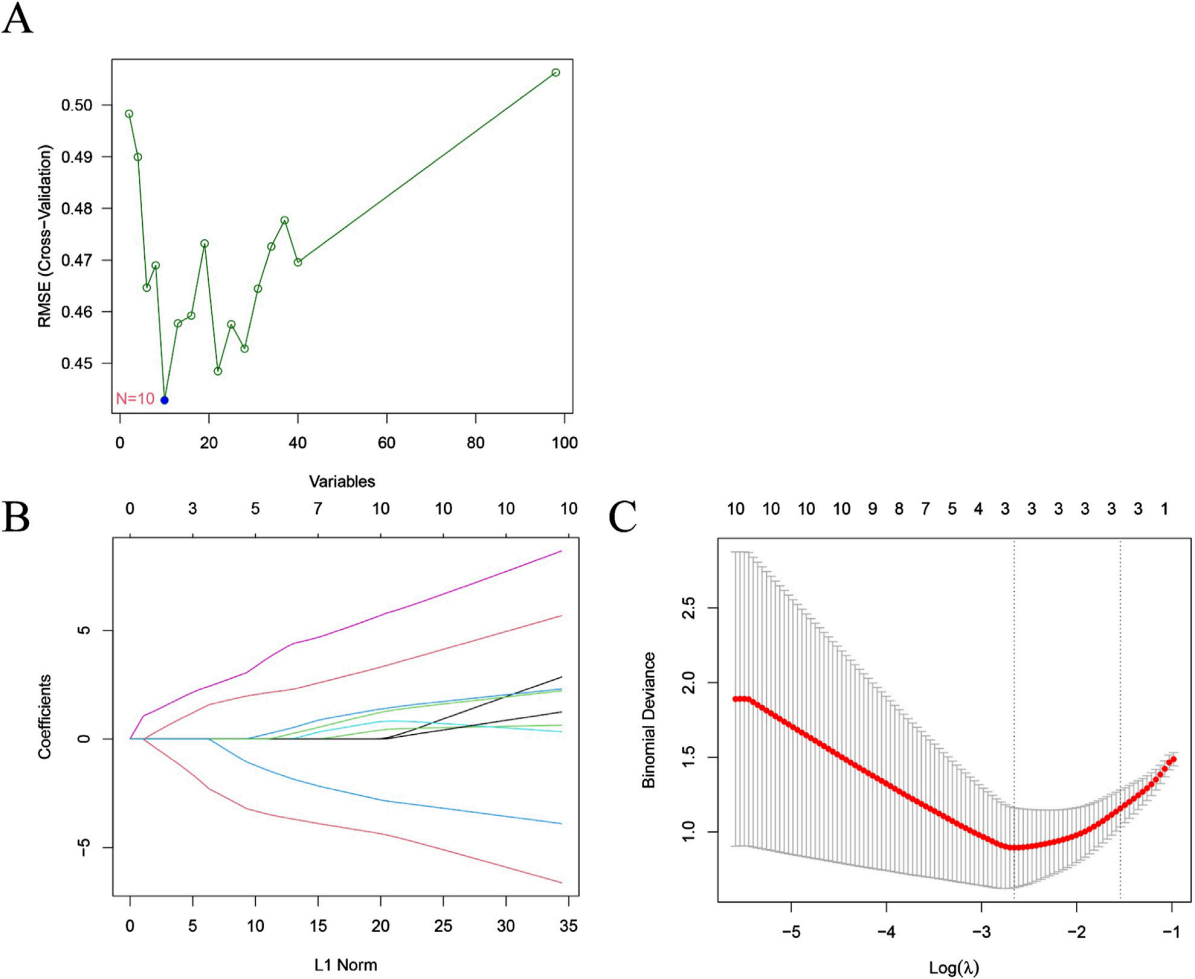
Features selection using machine learning algorithms. **(A)** Selection of characteristic genes via SVM-RFE algorithm; **(B,C)** Selection of characteristic genes via LASSO algorithm.

To identify hub genes with significant differences between HF patients and non-HF patients post-MI, we employed the SVM-RFE algorithm to select 10 candidate genes (*DZIP3*, *HIVEP2*, *ZNF366*, *CYLD*, *SNORD28*, *GPER1*, *E2F5, LHFPL2*, *ADAMTS2* and *ST14*) (Figure 2A). These genes were further refined via the LASSO algorithm, which identified three critical hub genes (*ZNF366*, *HIVEP2*, and *E2F5*) from an initial set of 98 common genes (Figures 2B,C). The 10 candidate genes were integrated for subsequent analyses (Supplementary Table S1).

**FIGURE 3:**
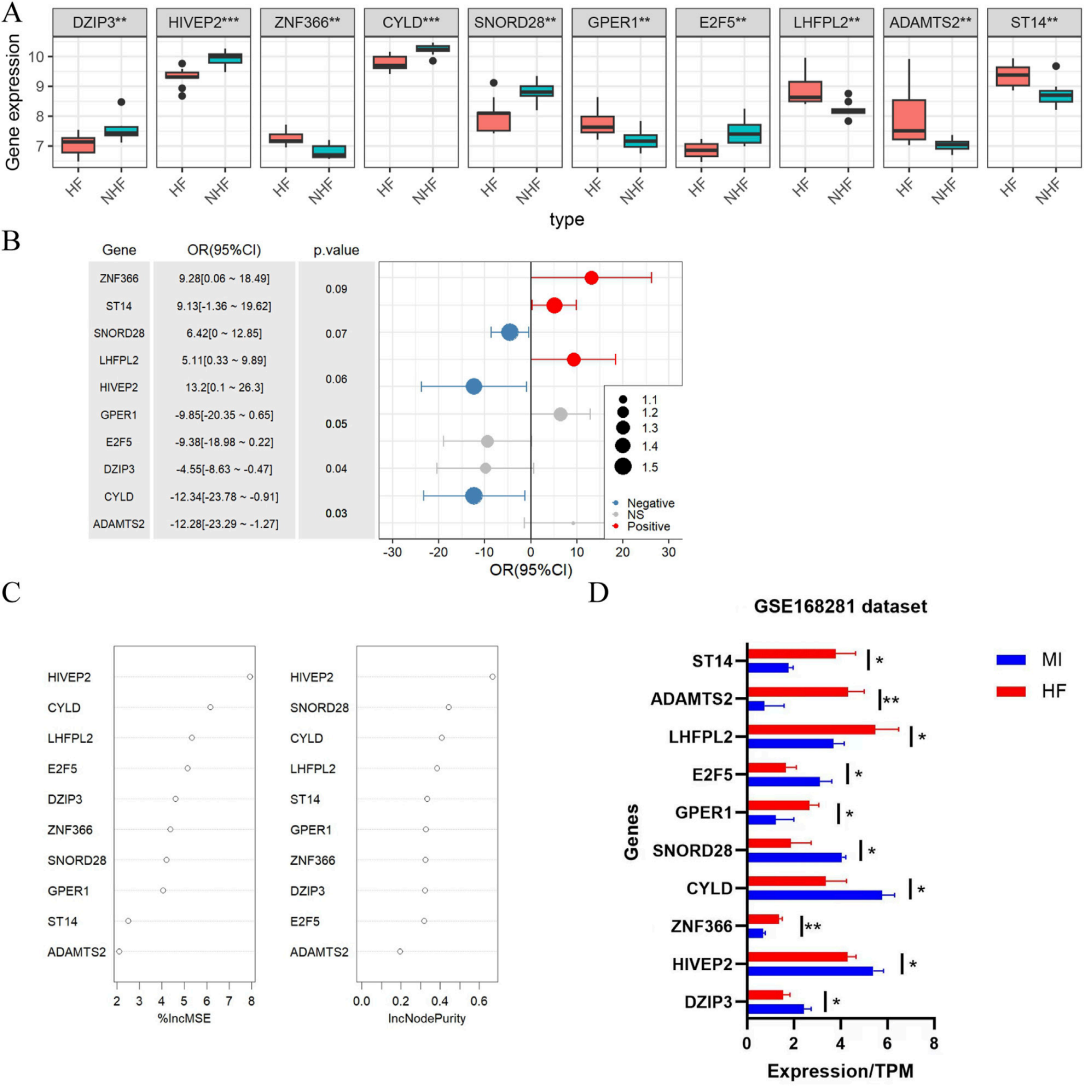
Differential expression and values of hub genes in the training and test groups. **(A)** Differential analysis of variables between HF patients and non-HF patients after MI; **(B)** Single-factor logistic regression analysis of variable associations with HF post-MI; **(C)** Feature importance assessment in the Random Forest model; **(D)** Expression levels of the 10 hub genes identified in GSE168281 dataset. Statistical significance was assessed using the Wilcoxon rank-sum test (two-sided). *P < 0.05, **P < 0.01.

Comparative analysis of hub gene expression revealed distinct patterns between HF patients and non-HF patients post-MI. *DZIP3*, *HIVEP2*, *CYLD*, *SNORD28* and *E2F5* were significantly downregulated in HF patients compared with non-HF patients (Figure 3A). Among these genes, *ZNF366*, *ST14* and *LHFPL2* were positively correlated with MI-to-HF progression, whereas *SNORD28*, *HIVEP2* and *CYLD* were negatively correlated (Figure 3B). Notably, *HIVEP2* and *CYLD* demonstrated the greatest functional importance in this progression (Figure 3C). To validate these findings, we analysed the GSE168281 dataset, which confirmed consistent expression trends and statistical significance (*P* < 0.05) (Figure 3D), supporting the reliability of our results.

### 3.3 Immune cell infiltration and correlation with hub genes in the MI-to-HF progression

**FIGURE 4:**
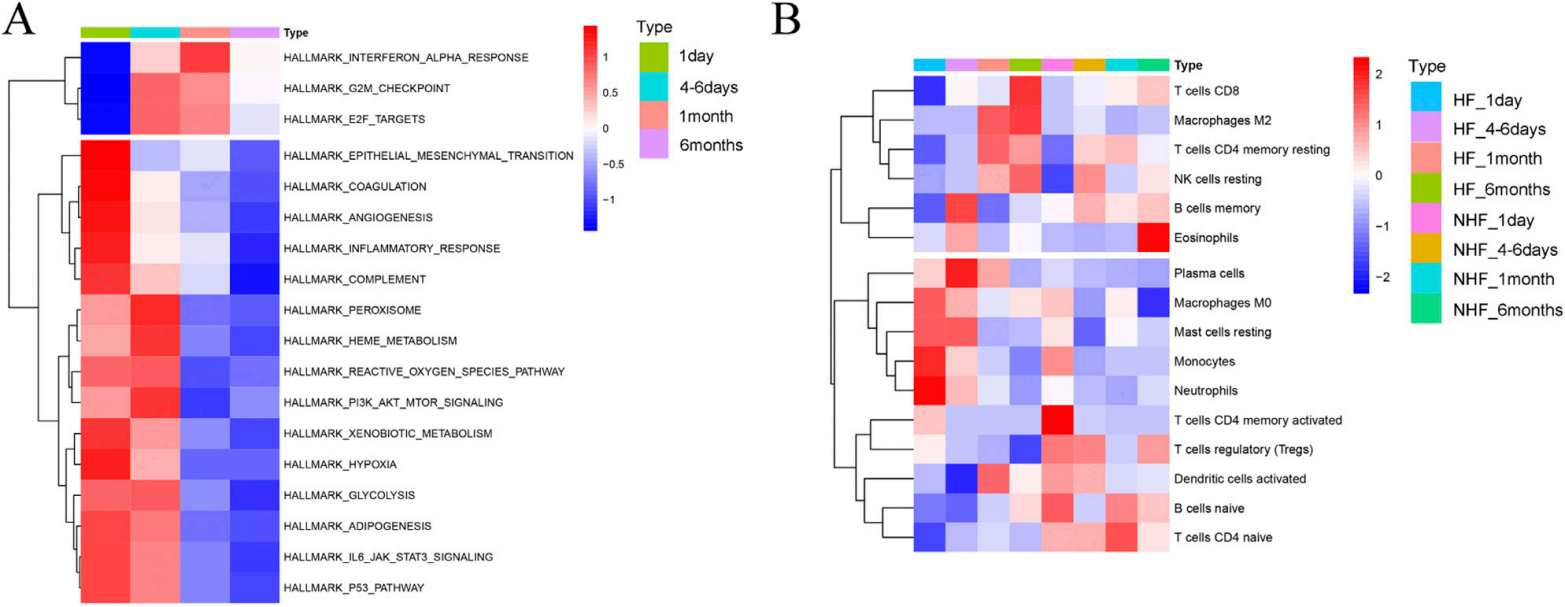
KEGG enrichment analysis for the progression from MI to HF. **(A)** Heatmap depicting the alteration of KEGG pathways in the procession from MI-to-HF; **(B)** Heatmap showing the alteration of immune characteristics in HF and non-HF after MI by CIBERSORT.

**FIGURE 5:**
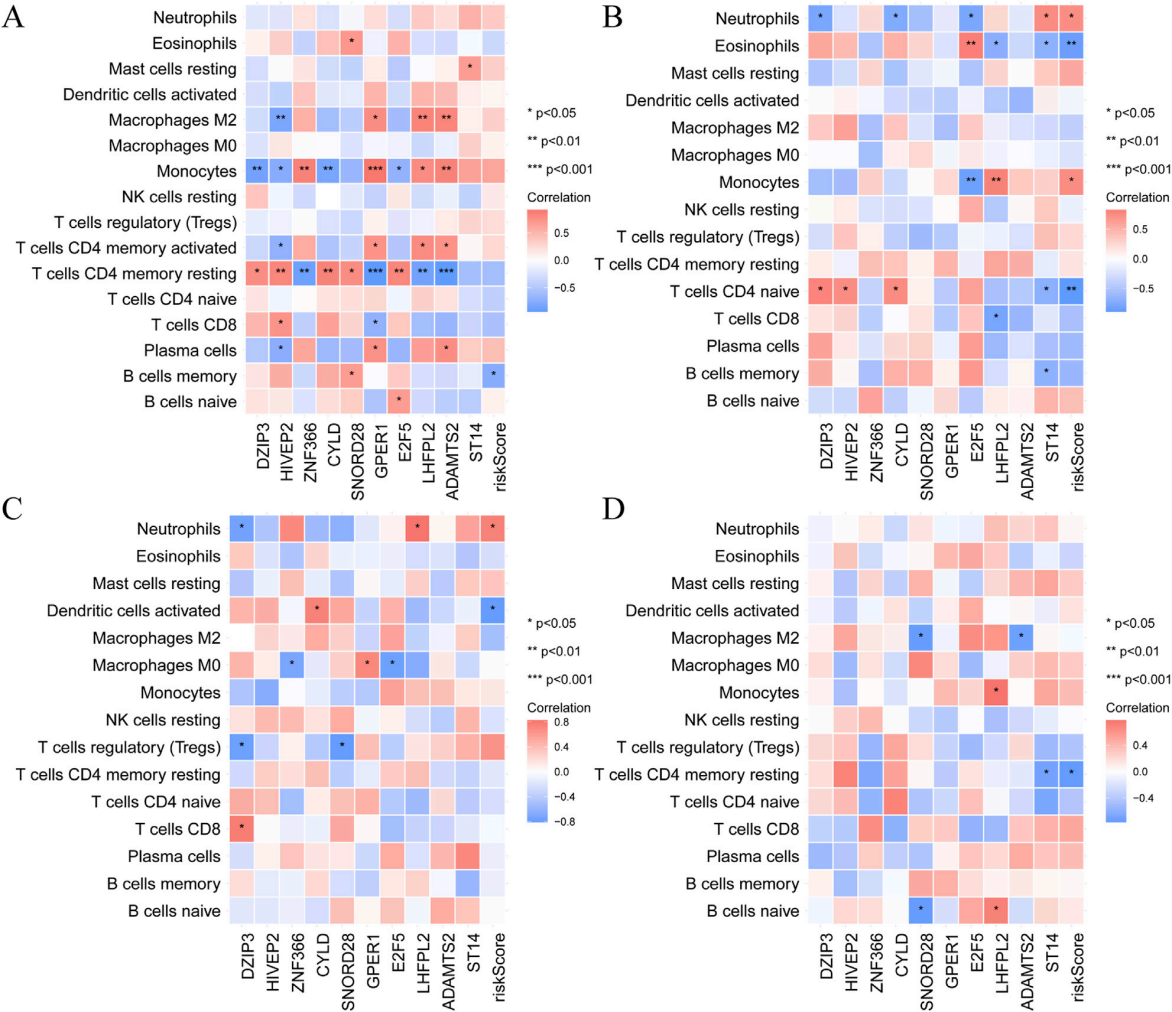
The relationship between hub gene expression and immune cell infiltration. **(A)** 1 days; **(B)** 4–6 days; **(C)** 30 days and **(D)** 180 days after MI.

To investigate the progression from MI to HF, we performed a KEGG enrichment analysis focusing on temporal changes in pathways following MI (Supplementary Figure S3A-F). Heatmaps revealed dynamic pathway alterations, highlighting key processes such as the inflammatory response, interferon alpha signalling, E2F targets, epithelial‒mesenchymal transition and glycolysis (Figure 4A). KEGG pathway analysis confirmed the involvement of immune-related pathways in the progression from MI to HF. Using CIBERSORT, we analysed immune cell infiltration and identified 16 immune cell subpopulations (Figure 4B). Compared with the non-HF post-MI group, the HF post-MI group exhibited significant dynamic changes in dendritic cells (DCs) and naïve CD4^+^ T cells. Hub genes, including *CYLD* and *HIVEP2*, exhibited strong correlations with immune cells in the HF group 1 day post-MI. These correlations persisted for most hub genes up to 6 days post-MI, except for *ZNF366*, *SNORD28*, *GPER1,* and *ADAMTS2*. At 30 days post-MI, the hub genes (excluding *HIVEP2*, *ADAMTS2* and *ST14*) maintained close associations with immune cells. Notably, *SNORD28*, *LHFPL2*, *ADAMTS2* and *ST14* maintained their immune cell correlations 180 days post-MI (Figure 5).

### 3.4 Construction and evaluation of machine learning models

We performed a rigorous comparative evaluation of machine learning algorithms for post-MI HF progression risk prediction, employing feature subsets derived from two distinct selection methodologies: SVM-RFE (Table 1) and LASSO (Table 2). Among the eight clinically relevant classifiers evaluated—LR, LDA, KNN, SVM, ridge regression, elastic net, NBC and XGBoost—the RF ensemble learning architecture demonstrated statistically significant superiority across four critical clinical performance metrics: sensitivity, specificity, positive predictive value and negative predictive value. This optimized RF implementation was consequently designated the principal predictive framework for subsequent mechanistic investigations of post-MI HF progression dynamics based on its balanced performance in terms of both statistical accuracy and clinical interpretability.

**TABLE 1:** Predictive model performance for HF progression risk using SVM-RFE features.

Term	Training cohort (1 day after MI)					Test cohort (4 days after MI)				
	AUC	Sensitivity	Specificity	PPV	NPV	AUC	Sensitivity	Specificity	PPV	NPV
Logistic Regression	1.000	1.000	1.000	1.000	1.000	0.528	0.222	1.000	0.500	0.364
Linear Discriminant Analysis	1.000	1.000	1.000	1.000	1.000	0.704	0.889	0.500	0.750	0.455
KNN	0.938	0.875	1.000	1.000	0.900	0.750	0.500	1.000	1.000	0.750
SVM	0.938	0.875	1.000	1.000	0.900	0.778	0.667	0.889	0.800	0.800
Random Forest	1.000	1.000	1.000	1.000	1.000	0.926	0.889	0.833	1.000	0.545
Ridge	0.986	1.000	0.875	0.900	1.000	0.759	0.778	0.667	0.667	0.417
Elastric Net	1.000	1.000	1.000	0.900	1.000	0.741	0.889	0.667	0.667	0.417
Naïve Bayes Classifier	0.826	0.708	0.806	0.875	0.778	0.667	0.333	1.000	1.000	0.500
Xgboost	1.000	1.000	1.000	1.000	1.000	0.889	1.000	0.833	1.000	0.500

**TABLE 2:** Predictive model performance for HF progression risk using LASSO features.

Term	Training cohort (1 day after MI)					Test cohort (4 days after MI)				
	AUC	Sensitivity	Specificity	PPV	NPV	AUC	Sensitivity	Specificity	PPV	NPV
Logistic Regression	0.986	1.000	0.875	0.889	0.875	0.796	0.778	0.833	0.800	0.500
Linear Discriminant Analysis	0.986	1.000	0.875	0.875	0.778	0.815	0.889	0.667	1.000	0.500
KNN	0.938	0.875	1.000	1.000	0.900	0.722	0.611	0.733	0.667	0.778
SVM	0.882	0.875	0.889	0.875	0.889	0.667	0.625	0.664	0.571	0.750
Random Forest	1.000	1.000	1.000	1.000	1.000	0.787	1.000	0.500	1.000	0.462
Ridge	0.986	1.000	0.875	0.875	0.778	0.833	0.889	0.667	1.000	0.500
Elastric Net	0.986	1.000	0.875	0.875	0.778	0.815	0.889	0.667	1.000	0.500
Naïve Bayes Classifier	0.826	0.778	0.875	0.875	0.778	0.611	0.222	1.000	1.000	0.462
Xgboost	0.001	0.001	0.001	0.001	0.889	0.796	0.667	0.833	1.000	0.462

**FIGURE 6:**
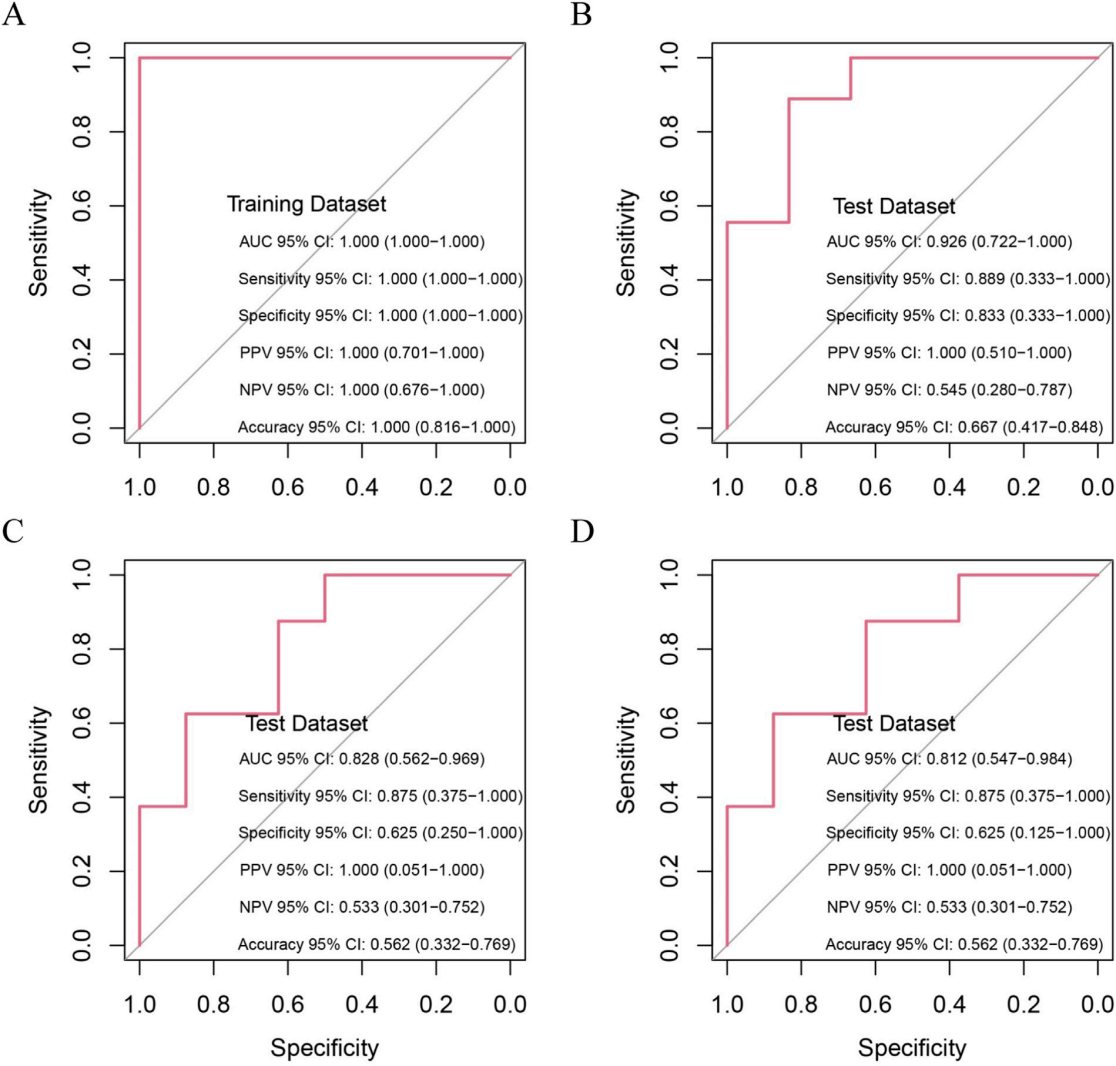
Prognostic Capability of Random Forest Model in Predicting HF Post-MI. **(A)** Ten hub genes distinguish MI patients at risk of HF from non-progressors within 1 day post-MI (training cohort); **(B–D)** Prognostic accuracy of hub genes in test cohort at 4–6 days, 1 month and 6 months post-MI.

To validate the diagnostic potential of the 10 candidate hub genes, we conducted rigorous ROC curve analyses using independent validation cohorts. The biomarker panel demonstrated exceptional acute-phase discrimination capacity, achieving perfect classification (AUC = 1.000, 95% CI 1.000-1.000) at 24 h post-MI in the training cohort (Figure 6A). Temporal performance analysis revealed peak diagnostic accuracy during the subacute phase (four to six days post-MI; AUC = 0.926, 95% CI 0.722–1.000), followed by gradual attenuation at chronic timepoints: 0.828 (95% CI 0.562–0.969) at 1 month and 0.812 (95% CI 0.547–0.984) at 6 months postinfarction (Figure 6B‒D). This temporal performance pattern suggests strong utility for early-stage heart failure detection in acute MI settings while highlighting the potential need for complementary biomarkers in chronic phase monitoring.

**FIGURE 7:**
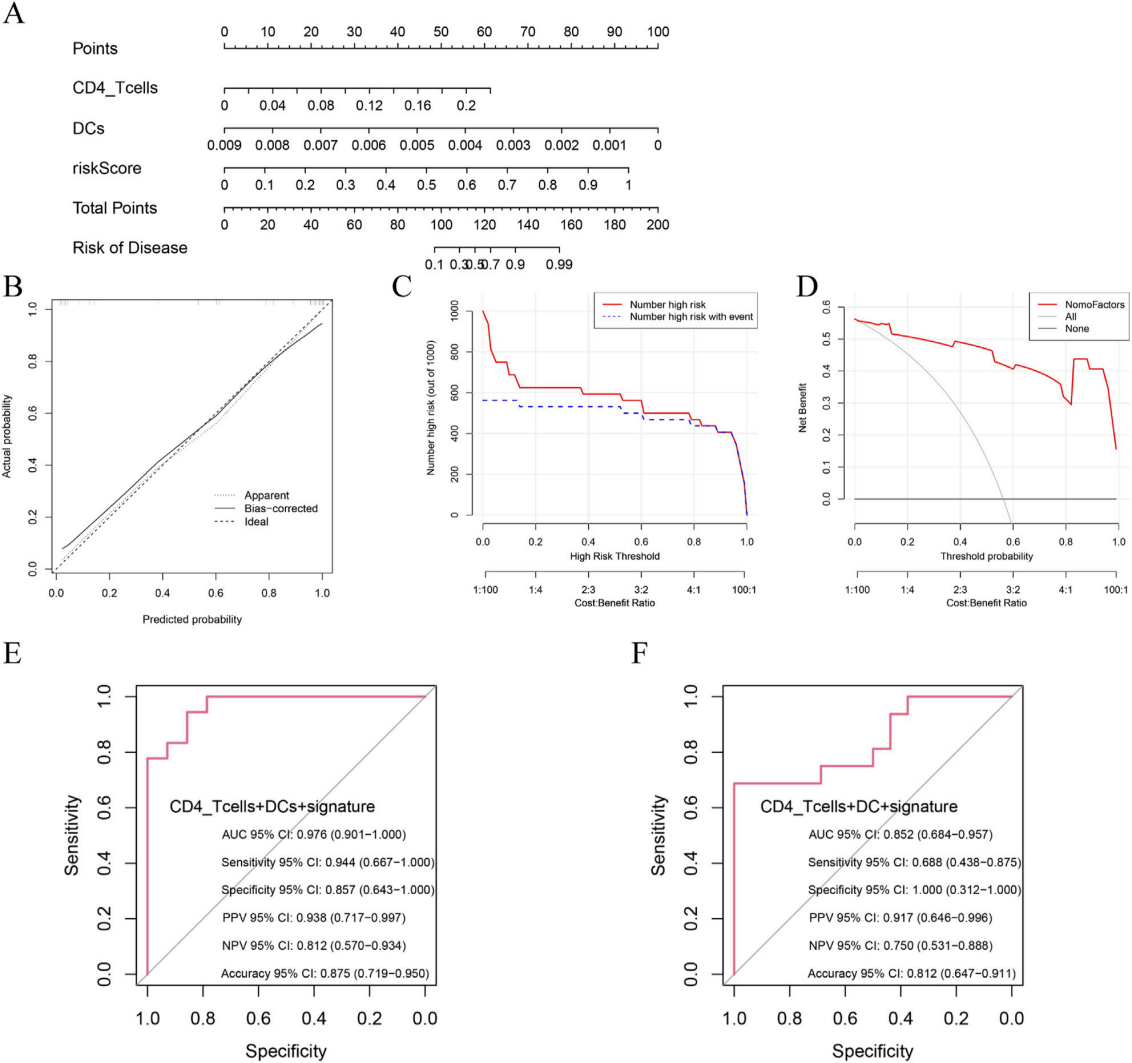
Prognostic performance of combined signatures in predicting HF progression post-MI. **(A)** Nomogram model integrating riskScore, DCs and CD4^+^T cells; **(B)** Calibration plot assessing model accuracy by comparing predicted and observed probabilities; **(C)** Decision curve analysis: identification of high-risk individuals at varying thresholds; **(D)** Decision curve analysis: net benefit across threshold probabilities; **(E)** Prognostic performance of combined signatures in predicting non-HF progression post-MI; **(F)** Prognostic performance of combined signatures in predicting HF progression post-MI.

We developed a multivariate prognostic nomogram integrating three critical components, (1) baseline risk score, (2) DCs abundance and (3) naïve CD4^+^ T cell frequency, with each parameter assigned weighted scores based on multivariate Cox regression coefficients (Figure 7A). The model demonstrated excellent calibration fidelity and clinical utility across the validation cohorts (Figures 7B–D). Notably, the combined model incorporating DCs, naïve CD4^+^ T cells, and hub gene expression profiles achieved exceptional diagnostic accuracy in non-HF patients (AUC = 0.976, 95% CI 0.901–1.000) and maintained robust performance in established HF patients (AUC = 0.852, 95% CI 0.684–0.957) (Figure 7E‒F). Comparative analysis demonstrated significant predictive improvement when immune cell biomarkers were combined with genomic signatures *versus* individual modalities (Supplementary Figure S4).

The superior predictive capacity of this immune‒genomic combination model may reflect the dual role of DC-mediated inflammatory responses and CD4^+^ T cell-regulated adaptive immunity in post-MI ventricular remodelling. Future single-cell sequencing studies could elucidate the spatial‒temporal interplay between these immune subsets and myocardial gene expression patterns during HF progression.

### 3.5 Single-cell transcriptomic profiling of the hub genes

**FIGURE 8:**
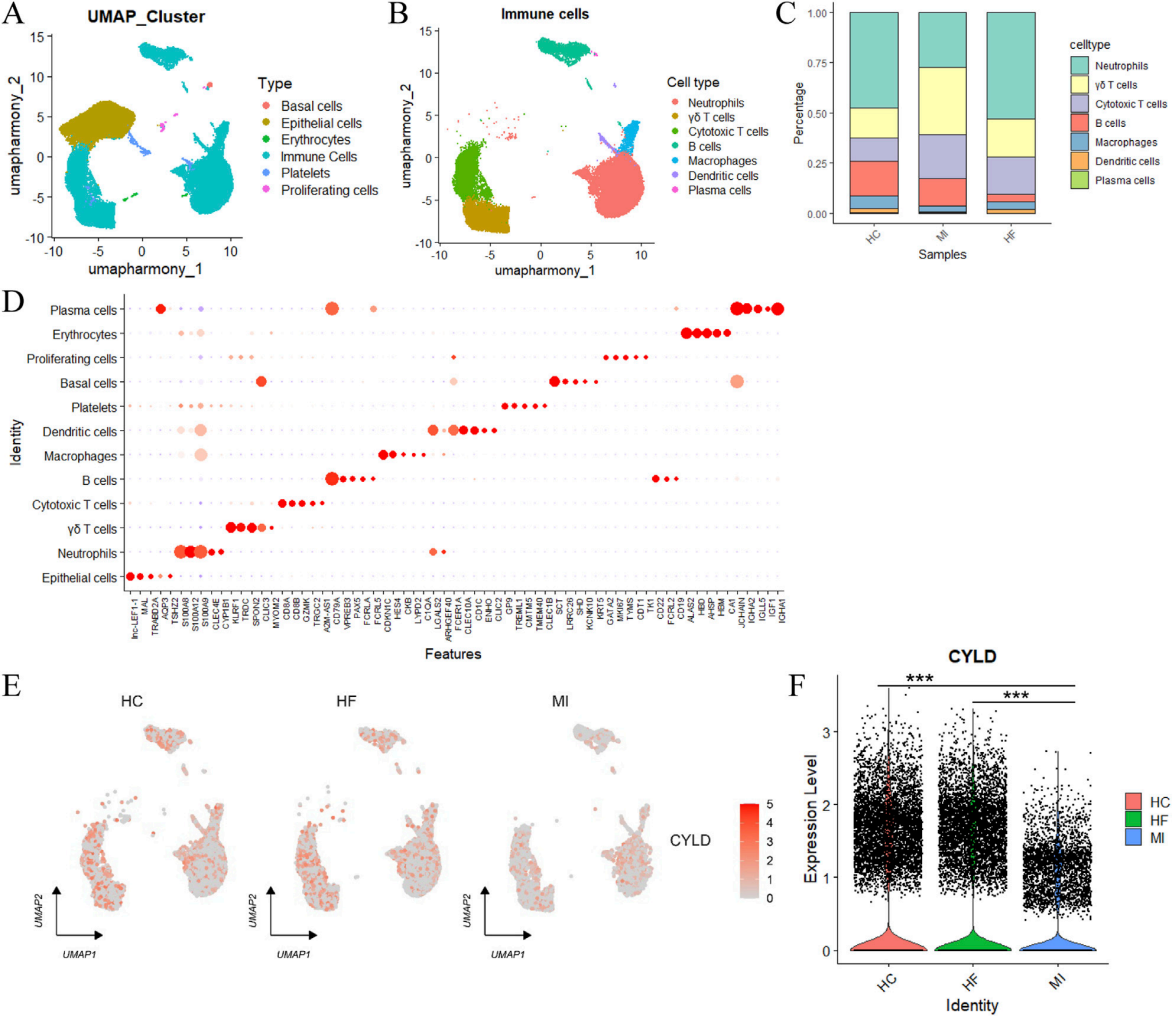
Single-cell analysis of cell populations. **(A)** UMAP visualization of annotated cell types; **(B)** Subclustering and annotation of immune cells; **(C)** The relative proportions of immune cell subtypes; **(D)** Feature plots used for cell type annotation; **(E)** UMAP distribution of target gene *CYLD* expression across groups; **(F)** Quantitative analysis of *CYLD* expression levels. Statistical significance was assessed using the Wilcoxon rank-sum test (two-sided). ***P < 0.001.

To elucidate the cellular specificity of the hub genes, we performed integrated analysis of single-cell RNA sequencing (scRNA-seq) data from two independent myocardial infarction cohorts (GSE226794 and GSE269269). Following batch effect correction via the Harmony integration algorithm (Supplementary Figure S5), we identified six major cellular compartments: basal cells, epithelial cells, erythrocytes, immune cells, platelets and proliferating cells (Figure 8A). Immune cell subclustering revealed seven functionally distinct subtypes, including neutrophils, γδ T cells, cytotoxic T cells, B cells, macrophages, DCs and plasma cells, with substantial compositional shifts between the MI and HF states (Figures 8B–D).

Hub gene expression mapping revealed cell type-specific enrichment patterns: *CYLD* exhibited predominant expression in immune subsets, whereas *SNORD28* was excluded from further analysis due to a lack of cell type specificity (Supplementary Figure S6). Strikingly, *CYLD* expression was markedly downregulated in HF-derived immune cells compared with that in MI samples (Figures 8E,F).

### 3.6 Functional and diagnostic validation of the *CYLD*

**FIGURE 9:**
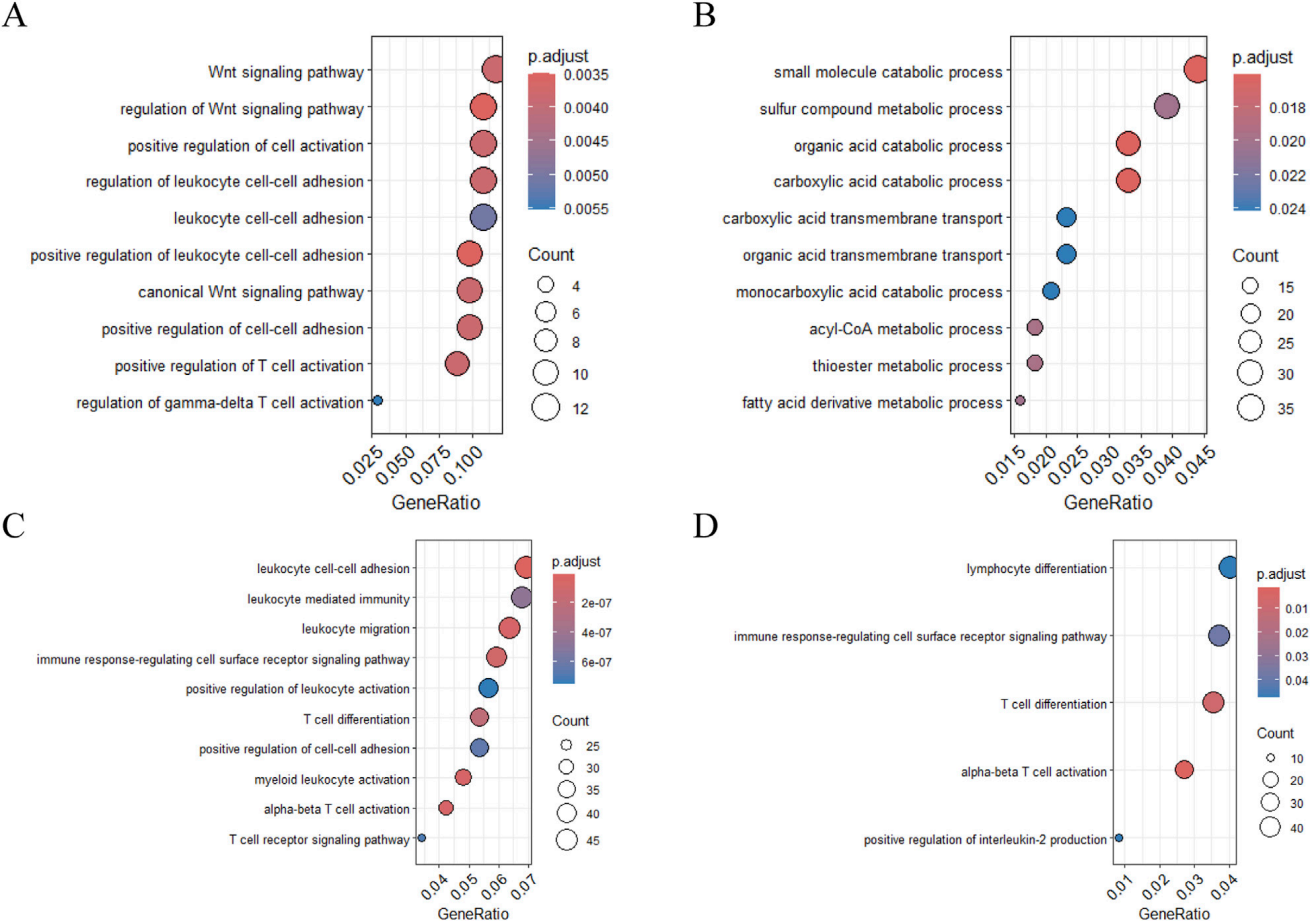
Functional enrichment analysis of target genes in T cells and B cells. **(A–B)** GO enrichment analysis of *CYLD* in T cells: **(A)** MI group and **(B)** HF group; **(C–D)** GO enrichment analysis of *CYLD* in B cells: **(C)** MI group and **(D)** HF group.

Gene Ontology (GO) enrichment analysis of *CYLD*-associated pathways revealed differential immune regulation between the MI and HF microenvironments. In MI samples, *CYLD* ^+^ T cells were enriched in positive regulation of T-cell activation and the Wnt signalling pathway (Figure 9A), whereas HF-associated *CYLD* ^+^ T cells were enriched in small molecule catabolic processes (Figure 9B). A similar dichotomy was observed in B-cell populations, with MI samples showing leukocyte cell‒cell adhesion *versus* HFs exhibiting lymphocyte differentiation (Figure 9C‒D).

The diagnostic potential of *CYLD* was validated through receiver operating characteristic (ROC) analysis (AUC = 0.817, 95% CI 0.647–0.940) in an independent validation cohort (Supplementary Figure S7). *CYLD* is a novel biomarker reflecting immune dysregulation.

## 4 Discussion

Despite therapeutic advances, post-MI HF remains a critical clinical challenge, with 45% of cases emerging within the first year postevent (Carberry et al., 2024). While early mortality rates have halved since the 1990s, chronic HF development continues to increase mortality risk compared with that of non-HF counterparts (Docherty et al., 2023; Carberry et al., 2024). These findings underscore the importance of early predictive biomarkers. We report that markers of HFs from MIs can be identified and that an HF warning can be issued.

Through integrative bioinformatics combining differential expression analysis and WGCNA, we identified 98 candidate genes linked to post-MI HF. By utilizing the machine learning algorithms LASSO and SVM-RFE, we successfully identified hub genes, namely, *GPER1*, *E2F5*, *DZIP3*, *CYLD*, *ADAMTS2*, *ZNF366*, *ST14*, *SNORD28*, *LHFPL2* and *HIVEP2*, from a panel of 98 candidate genes.

By utilizing a random forest model, which has proven to be highly effective, we were able to successfully predict the progression of MI patients to HF. Notably, the hub genes demonstrated robust predictive accuracy for HF subsequent to MI, maintaining consistent performance across diverse time points. This consistency suggests that these hub genes play critical roles in the underlying biological processes leading to HF development post-MI. However, with increasing time post-MI, the model’s predictions for HF become increasingly less precise. The progression to HF following MI is attributed to a multitude of complex pathological mechanisms, which encompass myocardial cell necrosis, inflammatory responses, fibrosis and the regulation of myocardial repair (Akhtar et al., 2024); therefore, this elevated level of uncertainty can be attributed to multiple factors, such as the intricacy of disease progression, alterations in patient health status over time, and the plausible interplay between diverse genetic and environmental factors. Nevertheless, our findings underscore the potential of hub genes as pivotal diagnostic tools for the early and precise identification of HF in patients with MI.

In our pathway enrichment analysis, we examined the alterations in pathways at various time points post-MI and their possible associations with the progression of HF. Notably, the inflammatory response plays a pivotal role in this context. Consistent with the findings of the present study, research has shown that individual variations in the magnitude of the inflammatory response following acute MI, which involves one or multiple inflammatory-modulating pathways, may play pivotal roles in promoting adverse left ventricular remodelling (Prabhu and Frangogiannis, 2016; Kufazvinei et al., 2024). This maladaptive process is closely associated with the progressive development of HF(Westman et al., 2016; Del Buono et al., 2022). Following MI, the resulting ischaemic injury elicits the mobilization and influx of a diverse spectrum of innate and adaptive immune cells to the infarcted myocardium, encompassing macrophages, DCs, T cells and various other cellular entities (Kretzschmar et al., 2012; Swirski and Nahrendorf, 2018; Jung et al., 2022; Delgobo et al., 2023). Simultaneously, inflammation plays a pivotal role in the progression of various aetiologies underlying HF(Carlisle et al., 2019; Paulus and Zile, 2021). Despite the mixed outcomes observed in clinical trials, the modulation of inflammation continues to hold promise as a target for therapeutic intervention in the management of HF(Riehle and Bauersachs, 2019). Thus, it can be inferred that immune cells play crucial roles in the progression of HF following MI.

Within the immunological axis, DCs, professional antigen-presenting cells expressing high levels of MHC class II, orchestrate post-MI adaptive immunity through three primary mechanisms: (1) neoantigen presentation via cross-dressing with cardiomyocyte-derived exosomes, (2) cytokine-mediated T-cell polarization, and (3) regulation of tertiary lymphoid structure formation in peri-infarct zones (Balan et al., 2019). Single-cell transcriptomics revealed that DC subpopulation dynamics correlate with HF progression rates, suggesting that DC activation states may serve as early prognostic indicators. Their activation status and functional proficiency within the post-MI milieu may critically influence the trajectory of HF progression. In addition to DCs, naïve CD4^+^ T cells are at the heart of adaptive immunity (Luckheeram et al., 2012). Inflammation plays a dual role in regulating the progression of HF post-MI by either promoting or inhibiting the processes of tissue repair and fibrosis in the injured myocardium (Lafuse et al., 2020; Wang et al., 2023).

*CYLD* is a specialized deubiquitinating enzyme with substrate specificity for lysine 63 (K63)- and methionine 1 (M1)-linked ubiquitin chains and exhibits minimal catalytic activity towards canonical K48-linked polyubiquitination marks critical for proteasomal degradation (Sato et al., 2015). Mechanistic studies have established its role as a master regulator of inflammatory signalling through targeted deubiquitination of key adaptor proteins, including NF-κB essential modulator (NEMO/IKKγ), TNF receptor-associated factors (TRAF2/6) and receptor-interacting protein kinase 1 (RIPK1), thereby attenuating both NF-κB and MAPK pathway activation (Brummelkamp et al., 2003; Kovalenko et al., 2003; Trompouki et al., 2003; Wright et al., 2007). Emerging evidence further implicates *CYLD* in Wnt/β-catenin signalling modulation via dishevelled (DVL) protein deubiquitination, suggesting broad-spectrum regulatory functions across developmental and oncogenic pathways (Tauriello et al., 2010; Fernandez-Majada et al., 2016). This pleiotropic enzyme orchestrates a delicate balance between proinflammatory responses and cellular homeostasis, with dysregulation implicated in pathological processes spanning chronic inflammation, immune dysregulation, and tumorigenesis (Marin-Rubio et al., 2023).

The pleiotropic nature of these biomarkers, particularly *CYLD’s* dual regulation of inflammatory and fibrotic pathways, positions them as promising candidates for both diagnostic stratification and targeted therapeutic intervention. The compartment-specific dysregulation of *CYLD*, a known regulator of T-cell receptor signalling and NF-κB activation, may underlie the transition from acute inflammatory responses to maladaptive immune remodelling during HF progression; its dual role in cardiomyocyte survival pathways and lymphocyte activation could explain its superior diagnostic performance compared with that of conventional myocardial stress markers.

Our findings indicate that the *CYLD* provides a more accurate prediction of the onset of HF after MI. However, our study has several limitations. Due to the challenges encountered in data collection and the practical difficulties associated with long-term patient follow-up for prognosis, our research results have not been validated in clinical patient samples. Furthermore, the mechanisms underlying the associations between these biomarkers and cellular targets in HF following MI remain to be fully elucidated. Future research should focus on understanding the biological pathways and interactions among these factors, as well as exploring potential therapeutic interventions that target these pathways.

## 5 Conclusion

In conclusion, our integrative multiomics investigation identified *CYLD* as a critical determinant of post-MI HF pathogenesis, serving a role as a prognostic biomarker. The *CYLD*-centred 10-gene signature enables early HF risk stratification with >80% accuracy post-MI, outperforming conventional biomarkers. Single-cell resolution reveals the immune-modulatory function of *CYLD*. The developed nomogram, which integrates *CYLD* expression, DC activation states and naïve CD4^+^ T cell counts, demonstrated superior predictive value. These findings fundamentally shift our understanding of post-MI, positioning *CYLD*-mediated ubiquitination as a linchpin mechanism governing the transition from MI to HF.

## Data Availability

The original contributions presented in the study are included in the article/Supplementary Material, further inquiries can be directed to the corresponding author.
