# Supplementary material for: CYLD as a key regulator of myocardial infarction-to-heart failure transition revealed by multi-omics integration
Source: Front Genet. 2025 Jun 23;16:1592985. doi: 10.3389/fgene.2025.1592985 (PMC12229883; doi:10.3389/fgene.2025.1592985)
Supplement: Supplementary file 1 [file Supplementaryfile1.docx]

Supplementary Material

# Supplementary Figures and Tables

## Supplementary Figures


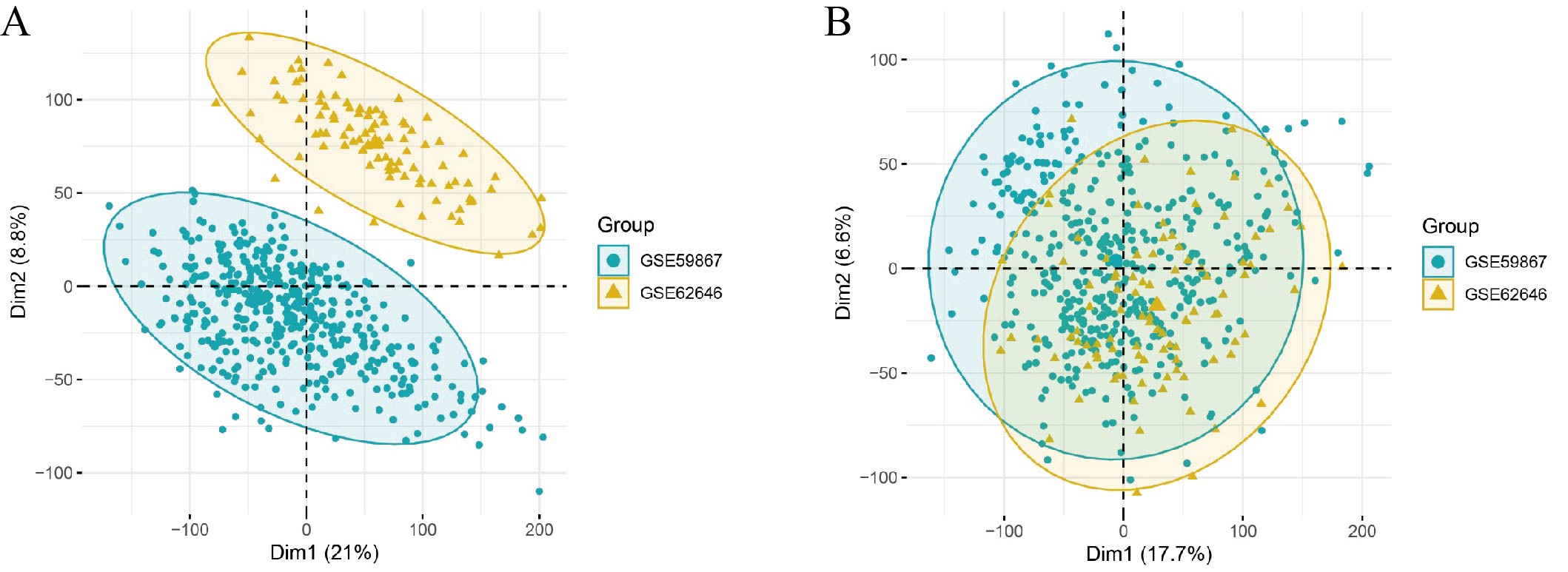


**Supplementary Figure 1.** Samples distribution in GSE59867 and GSE62646 datasets. (A-B) Distribution of samples before and after normalization, using “limma” package between two datasets.


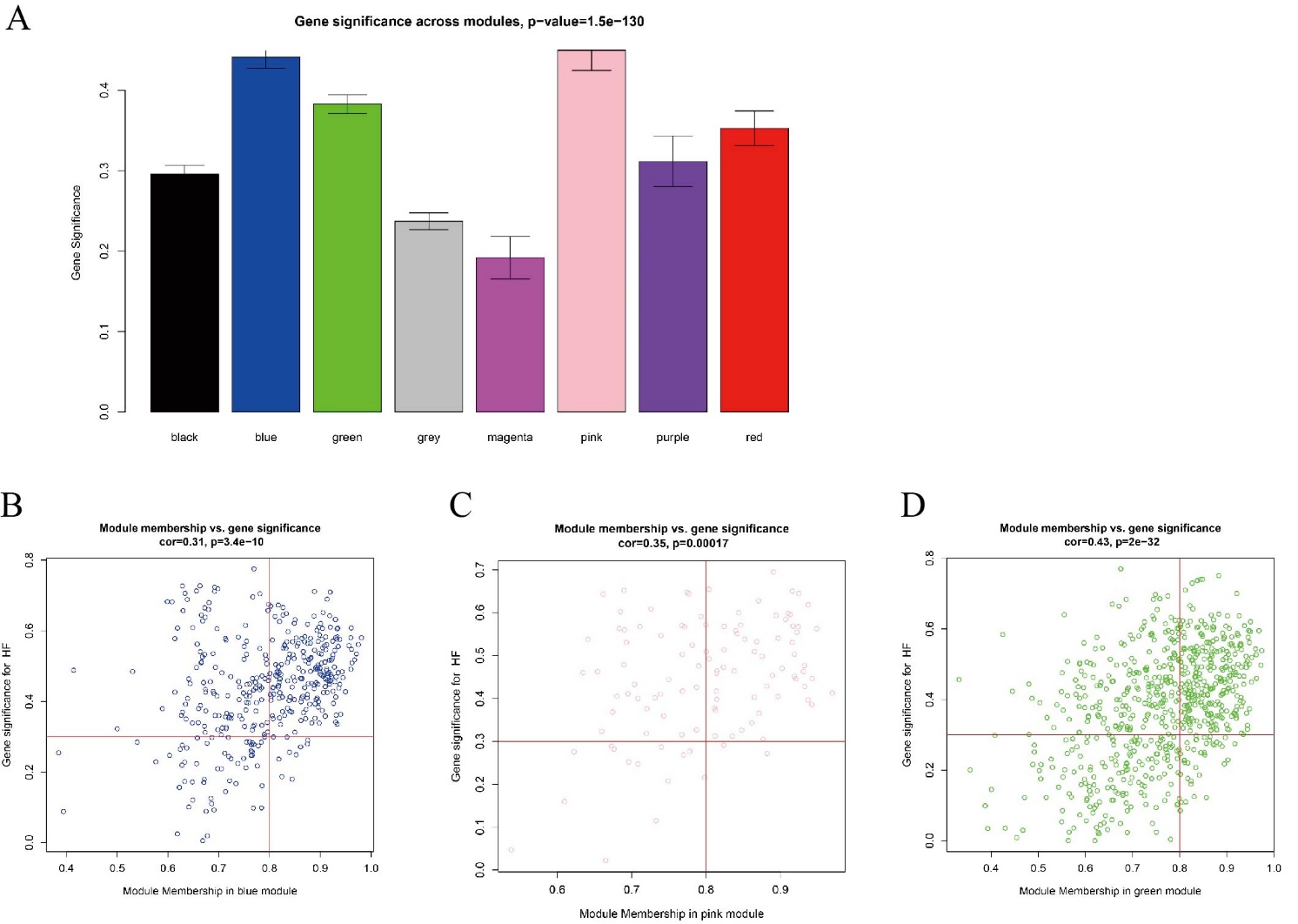


**Supplementary Figure 2.** Weighted gene co-expression network analysis. (A) Histogram implying the correlation between modules and the traits of MI patients at risk of progression to HF; (B-D) The scatter plots showing the association between module membership and gene significance in blue, pink and green modules for the genes.


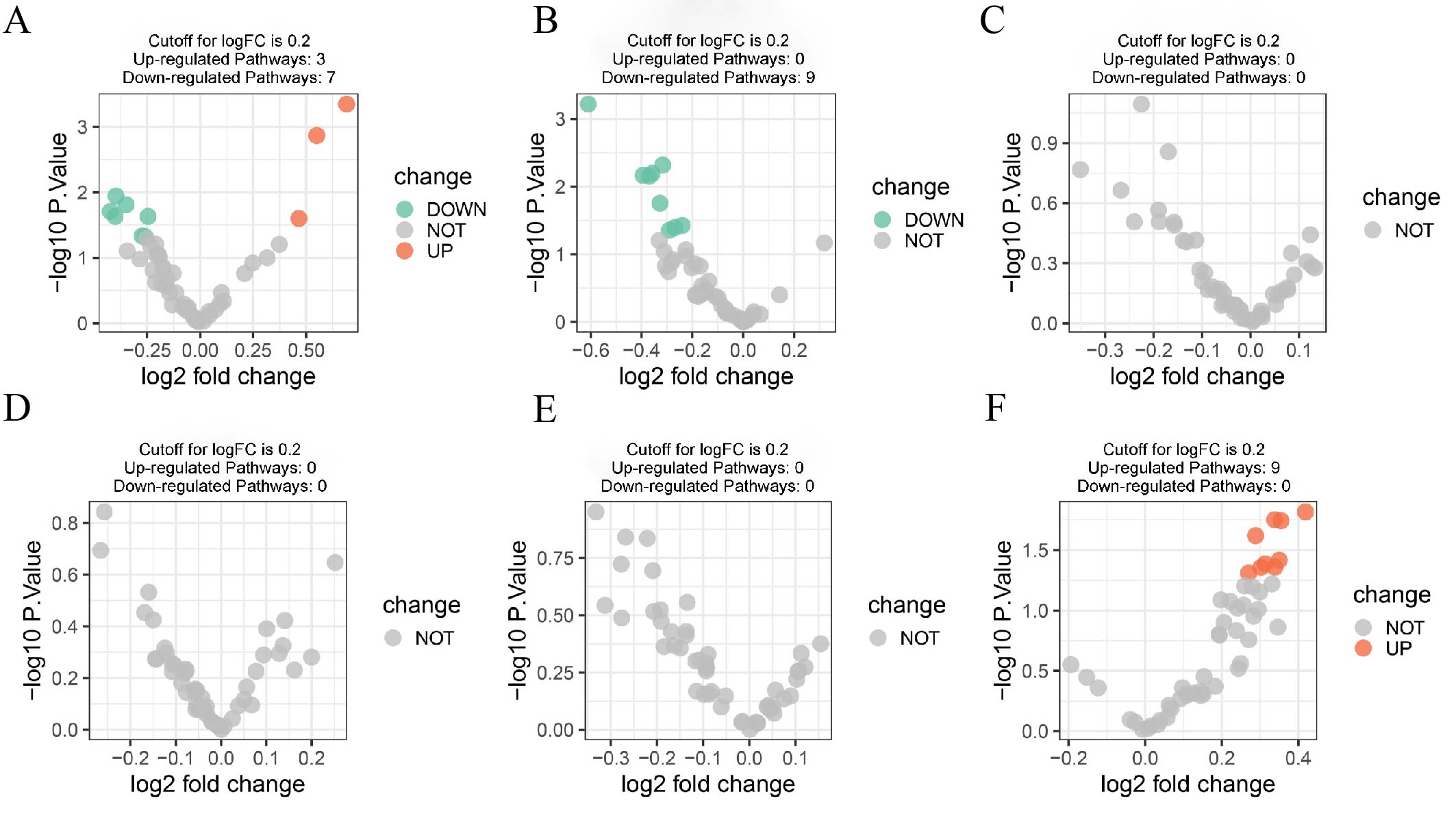


**Supplementary Figure 3.** KEGG Enrichment Analysis of Pathway lterations in the Progression from MI-to-HF. (A) Volcano plot comparing pathways at 4-6 days VS. 1 day post-MI diagnosis in HF post-MI patients; (B) Volcano plot depicting pathway differences between 30 days and 4-6 days post-MI diagnosis in HF post-MI patients; (C) Volcano plot showing pathway alterations from 180 days to 30 days in HF post-MI patients; (D) Volcano plot contrasting pathways at 4-6 days VS.1 day post-MI diagnosis in non-HF post-MI patients; (E) Volcano plot displaying pathway changes between 30 days and 4-6 days post-MI diagnosis in non-HF post-MI patients; (F) Volcano plot illustrating pathway alterations from 180 days to 30 days post-MI diagnosis in the non-HF post-MI patients.


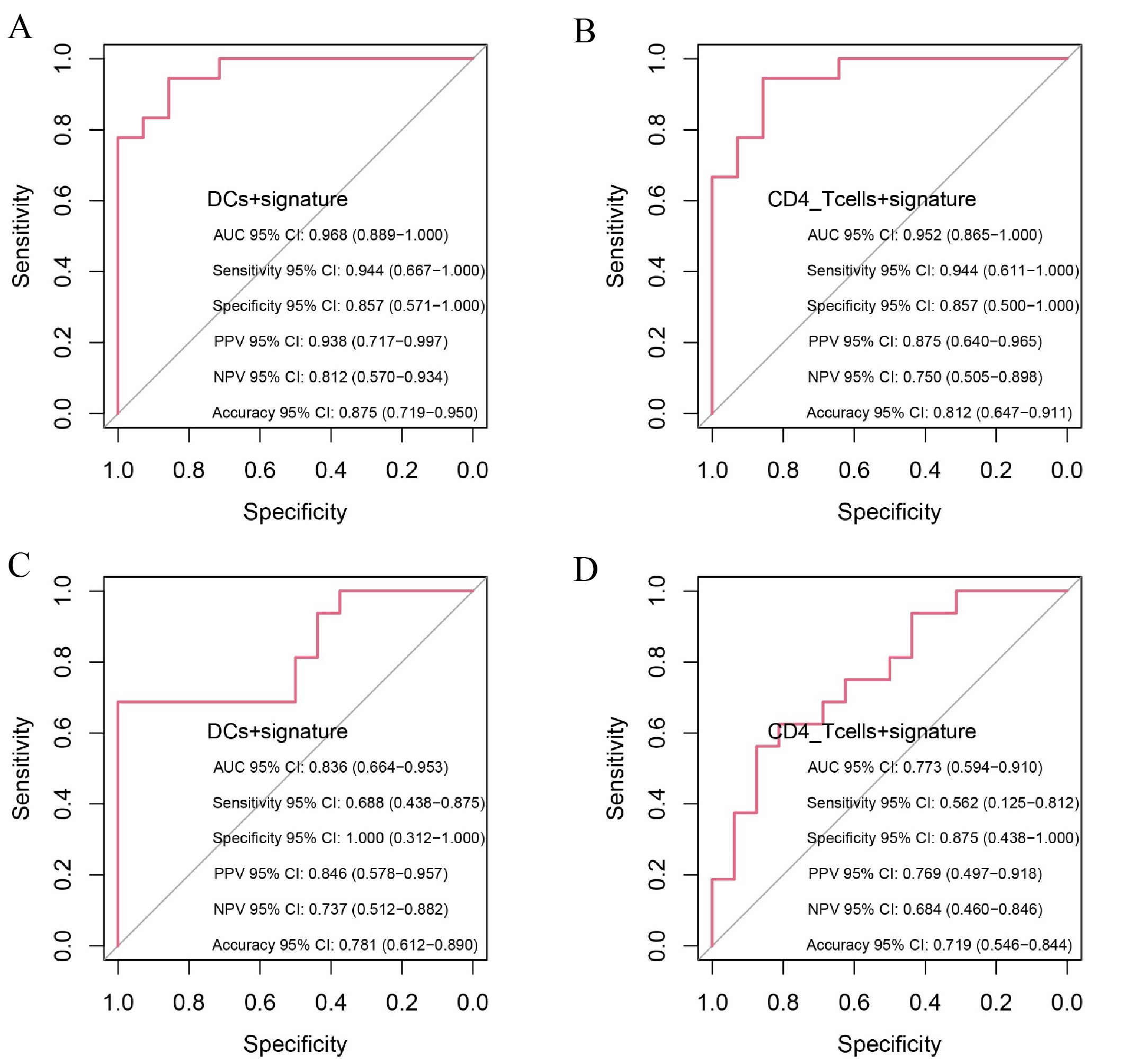


**Supplementary Figure 4.** Prognostic performance of combined signatures in predicting HF progression post-MI. (A-B) ROC curves and predictive accuracy in the non-HF group post-MI; (C-D) ROC curves and predictive accuracy in the HF group post-MI.


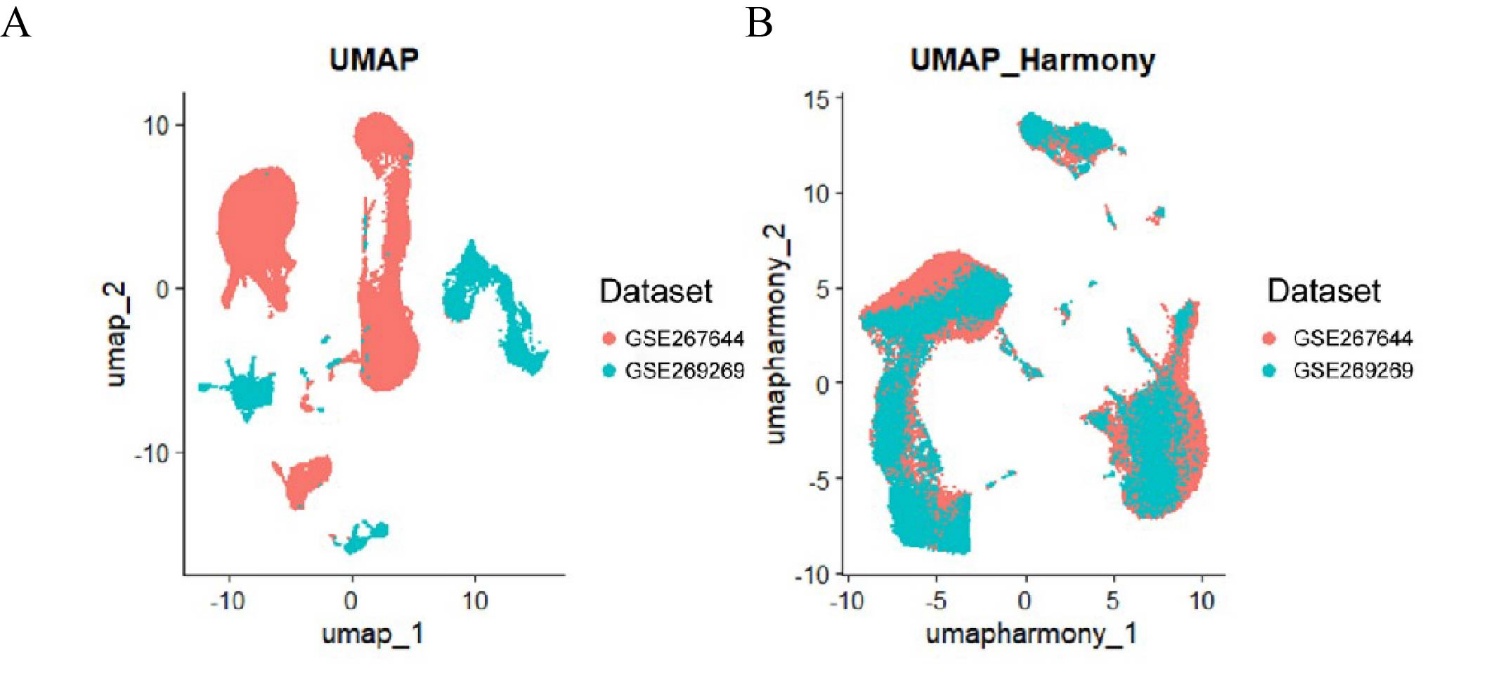
**Supplementary Figure 5.** Single-cell sequencing analysis. (A) Data visualization before batch effect removal; (B) Data visualization after batch effect removal using Harmony.


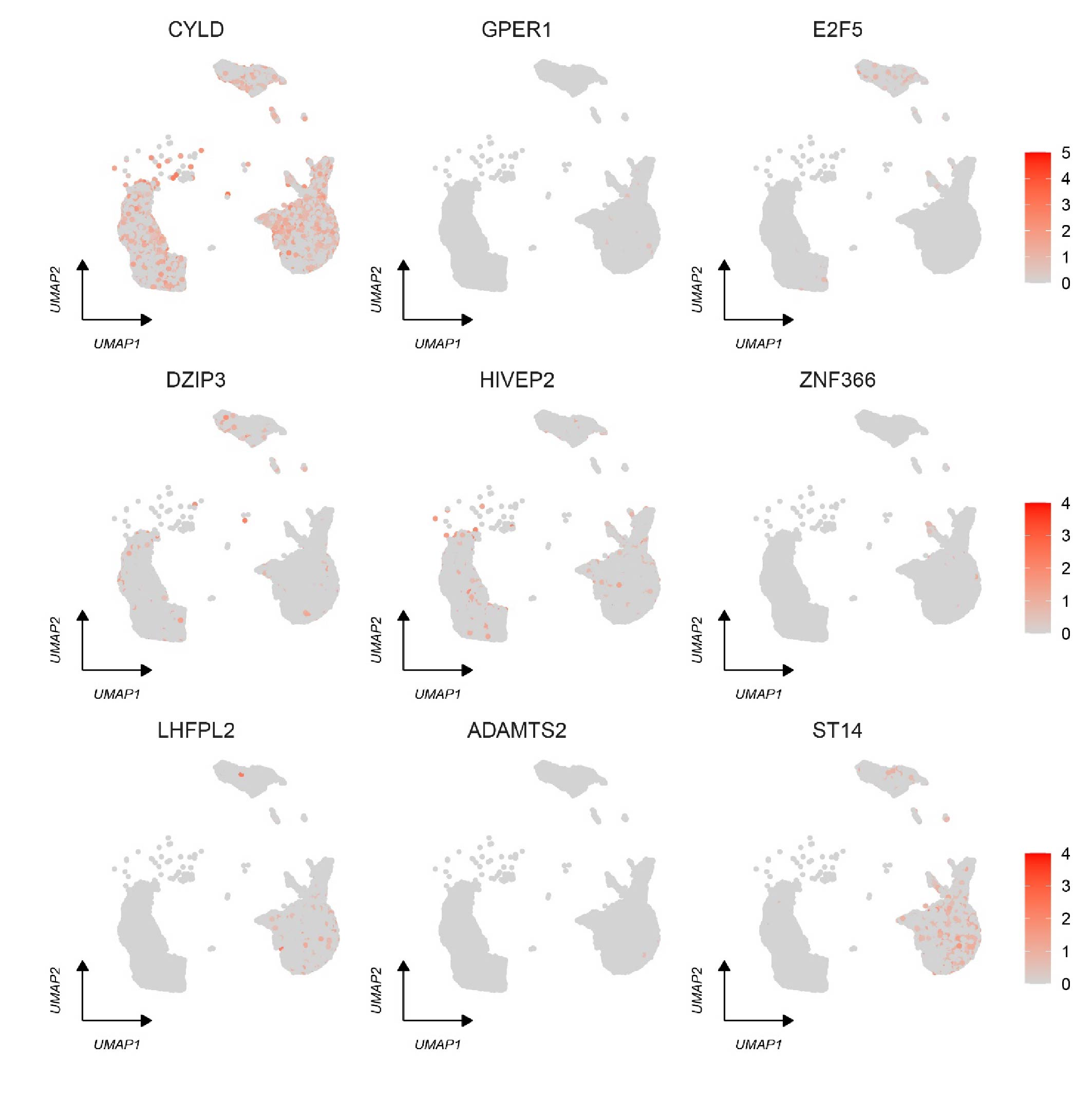


**Supplementary Figure 6.** Expression distribution of hub genes in immune cell populations.


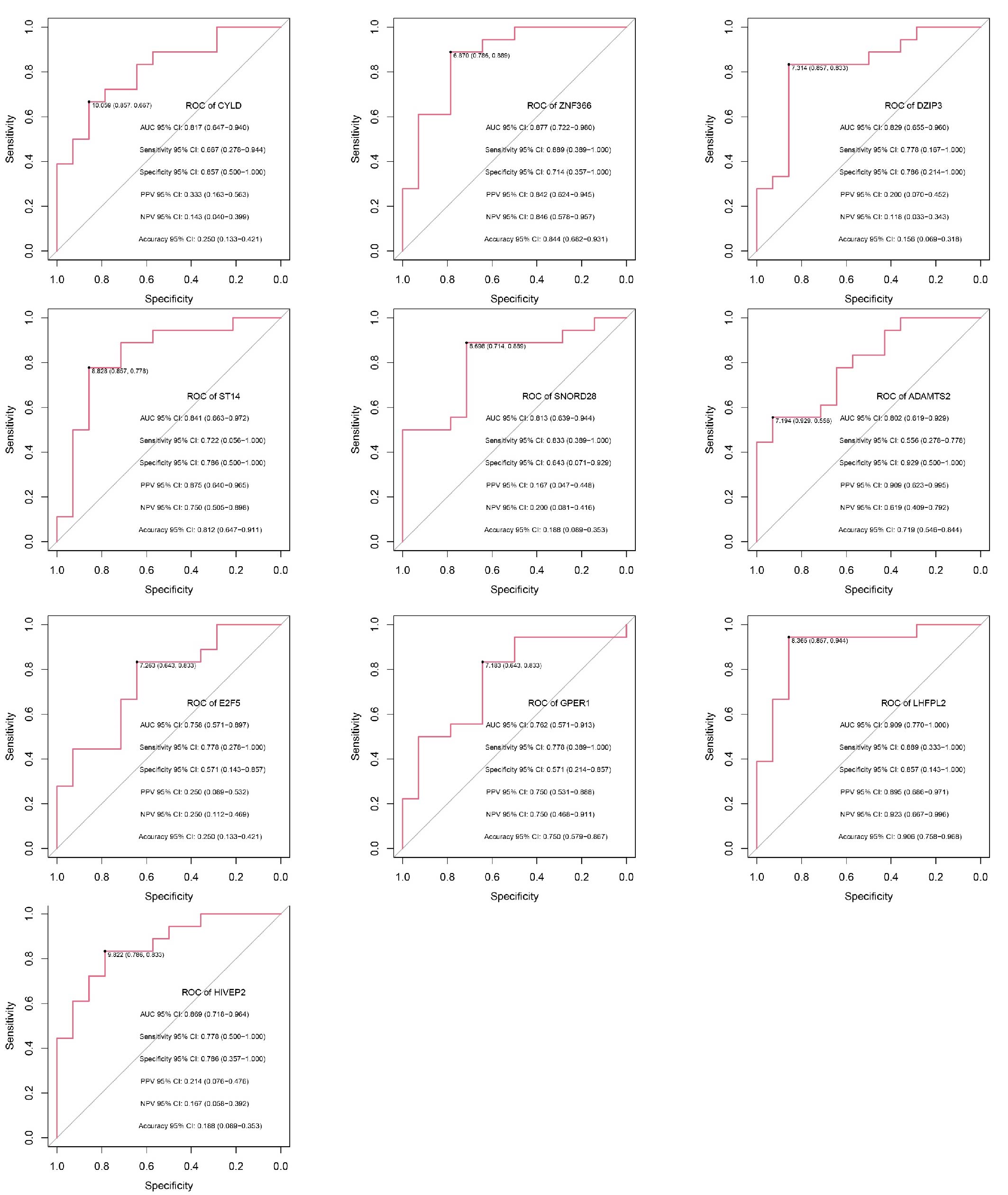


**Supplementary Figure 7.** AUC values of variables in predicting HF incidence in MI patients.

## Supplementary Table

Supplementary Table 1 The description of hub genes.

| Gene | Description |
| --- | --- |
| *ADAMTS2* | A Disintegrin And Metalloproteinase with Thrombospondin Motifs 2 |
| *CYLD* | CYLD Lysine 63 Deubiquitinase |
| *DZIP3* | DAZ Interacting Protein 3 |
| *E2F5* | E2F Transcription Factor 5 |
| *GPER1* | G Protein-Coupled Estrogen Receptor 1 |
| *HIVEP2* | HIVEP Zinc Finger Protein 2 |
| *LHFPL2* | Lipoma HMGIC Fusion Partner-Like 2 |
| *ST14* | Suppression Of Tumorigenicity 14 |
| *SNORD28* | Small Nucleolar RNA, C/D Box 28 |
| *ZNF366* | Zinc Finger Protein 366 |
